# Virulence Induction in Pseudomonas aeruginosa under Inorganic Phosphate Limitation: a Proteomics Perspective

**DOI:** 10.1128/spectrum.02590-22

**Published:** 2022-11-10

**Authors:** Miguel A. Matilla, Zulema Udaondo, Sandra Maaß, Dörte Becher, Tino Krell

**Affiliations:** a Department of Biotechnology and Environmental Protection, Estación Experimental del Zaidín, Consejo Superior de Investigaciones Científicas, Granada, Spain; b Department of Biomedical Informatics, University of Arkansas for Medical Sciences, Little Rock, Arkansas, USA; c Department of Microbial Proteomics, Institute of Microbiology, University of Greifswald, Greifswald, Germany; University Roma Tre

**Keywords:** *Pseudomonas aeruginosa*, phosphate starvation, proteomics, anti-infective therapy, pyocyanin, quorum sensing, virulence

## Abstract

Inorganic phosphate (Pi) is a central nutrient and signal molecule for bacteria. Pi limitation was shown to increase the virulence of several phylogenetically diverse pathogenic bacteria with different lifestyles. Hypophosphatemia enhances the risk of death in patients due to general bacteremia and was observed after surgical injury in humans. Phosphate therapy, or the reduction of bacterial virulence by the administration of Pi or phosphate-containing compounds, is a promising anti-infective therapy approach that will not cause cytotoxicity or the emergence of antibiotic-resistant strains. The proof of concept of phosphate therapy has been obtained using primarily Pseudomonas aeruginosa (PA). However, a detailed understanding of Pi-induced changes at protein levels is missing. Using pyocyanin production as proxy, we show that the Pi-mediated induction of virulence is a highly cooperative process that occurs between 0.2 to 0.6 mM Pi. We present a proteomics study of PA grown in minimal medium supplemented with either 0.2 mM or 1 mM Pi and rich medium. About half of the predicted PA proteins could be quantified. Among the 1,471 dysregulated proteins comparing growth in 0.2 mM to 1 mM Pi, 1,100 were depleted under Pi-deficient conditions. Most of these proteins are involved in general and energy metabolism, different biosynthetic and catabolic routes, or transport. Pi depletion caused accumulation of proteins that belong to all major families of virulence factors, including pyocyanin synthesis, secretion systems, quorum sensing, chemosensory signaling, and the secretion of proteases, phospholipases, and phosphatases, which correlated with an increase in exoenzyme production and antibacterial activity.

**IMPORTANCE** Antibiotics are our main weapons to fight pathogenic bacteria, but the increase in antibiotic-resistant strains and their consequences represents a major global health challenge, revealing the necessity to develop alternative antimicrobial strategies that do not involve the bacterial killing or growth inhibition. P. aeruginosa has been placed second on the global priority list to guide research on the development of new antibiotics. One of the most promising alternative strategies is the phosphate therapy for which the proof of concept has been obtained for P. aeruginosa. This article reports the detailed changes at the protein levels comparing P. aeruginosa grown under Pi-abundant and Pi-depleted conditions. These data describe in detail the molecular mechanisms underlying phosphate therapy. Apart from Pi, several other phosphate-containing compounds have been used for phosphate therapy and this study will serve as a reference for comparative studies aimed at evaluating the effect of alternative compounds.

## INTRODUCTION

Pseudomonas aeruginosa (PA) is among the most feared human pathogens. It is an opportunistic pathogen that frequently causes nosocomial disease by infecting debilitated patients. PA is omnipresent in the environment and has been detected in different soil, water, human and animal-derived samples, different foods like salads, vegetables, and milk, as well as in plumbing systems or hospital settings (1–3). PA is a highly versatile pathogen, able to infect almost all human tissues, including the respiratory tract, ear, eye, brain, heart, and urinary tract, and causes a general bacteremia (4). PA is of important clinical relevance since: (i) infections are associated with significant death rates; (ii) it is among the most frequent causes of nosocomial infections; and (iii) multidrug-resistant strains are rapidly emerging (5–8). In addition to humans, PA infects animals and plants (9, 10).

Phosphorous is an essential element for all living cells and required for the synthesis of ATP, nucleic acids, phospholipids, and other biomolecules. In addition to its metabolic importance, inorganic phosphate (Pi) is an important signal molecule that modulates virulence in many different pathogens, including human-pathogenic bacteria like Vibrio cholerae (11), Bacillus anthracis (12), Mycobacterium tuberculosis (13), Staphylococcus aureus (14), the bacterial phytopathogens Agrobacterium tumefaciens (15) and Xanthomonas oryzae (16), or the human fungal pathogen Candida albicans (17). In the context of PA, Pi starvation was found to elicit important transcriptional changes (18) that were shown to shift this bacterium toward virulent phenotypes (19–21). The role of Pi as a signal molecule that controls virulence is likely to be related to the low phosphate availability in a healthy person (1.25 mM) that then becomes limited in patients following chemotherapy or who have undergone a recent surgical intervention (<0.03 mM) (20–22).

Pi is sensed by a sophisticated signaling complex comprised by the PstABCS phosphate transporter that is linked through the coupling protein PhoU to the PhoRB two-component system. Under Pi-limiting conditions, the PhoR kinase phosphorylates its response regulator PhoB to regulate gene expression (23). Several studies show that Pi starvation causes a PhoRB-mediated activation of the Rhl and PQS quorum sensing (QS) systems, whereas the Las QS system is dispensable (24–26).

PA was found to be present in the intestine of 20% of healthy individuals and up to 50% of hospitalized patients (27). Furthermore, the intestinal tract was identified as the primary site from which PA spreads, causing sepsis (28). Surgical interventions were shown to deplete intestinal Pi, in turn causing an activation of bacterial virulence (20, 21). A mouse model was established in which surgical injury was induced followed by intestinal PA inoculation (21). Several studies showed that the oral administration of Pi prior to the surgical injury of mice protected the animals against infections caused by PA (21, 29) and C. albicans (30). This approach was termed phosphate therapy and subsequently optimized using different phosphate-containing polyethylene glycol (PEG) polymers combining the favorable properties of Pi and PEG (31–34) or the use of nanomaterials that release Pi (35). The administration of Pi-PEG polymers not only suppressed virulence in PA but also in Serratia marcescens, Klebsiella oxytoca, Enterococcus faecalis, Klebsiella oxytoca, C. albicans (31), and in multispecies communities isolated form the intestine (33). In addition, the application of polyphosphate also caused a reduction of S. marcescens and PA virulence treats (36). These data suggest a universal nature of phosphate therapy to fight different classes of pathogens.

The increase in antibiotic-resistant microorganisms and their consequences represents a major global health challenge. Antimicrobial-resistant infections kill 700,000 patients per year (37, 38) and estimations indicate that this toll will rise to 10 million deaths annually by 2050 (38). Due to several different reasons, the pipeline to develop novel antibiotics is drying out (39). The current situation is referred to as antibiotics resistance crisis (40) and the World Health Organization (WHO) warns that a postantibiotic era, in which common infection and minor injuries kill, is far from being an apocalyptic fantasy, but a real possibility (41). As a result, the WHO has rated the development of new antimicrobial agents as critical and has placed PA second on global priority list of antibiotic-resistant bacteria to guide research, discovery, and development of new antibiotics (42). However, fighting antibiotic-resistant bacteria with novel antibiotics will cause again the selection of resistant strains, resulting in a vicious cycle that may not permit to tackle antimicrobial resistance over the long term. Although there is a desperate need for the discovery of novel antibiotics, approaches like the phosphate therapy are promising alternatives to fight pathogenic microorganisms (43–46).

Comprehensive information on the changes at the protein level caused by Pi scarceness in PA is lacking. However, this information is essential to comprehend the molecular basis underlying phosphate therapy and it is the primary objective of this article to fill this gap in knowledge. We report here a proteomics study of PA grown in rich medium, or minimal medium supplemented with 0.2 mM or 1 mM Pi. About half of the predicted PA proteins could be quantified and vast changes at the protein level were detected comparing samples containing 0.2 mM and 1 mM Pi.

## RESULTS AND DISCUSSION

### Pyocyanin production is stimulated over a very narrow Pi window in a highly cooperative process.

**FIG 1:**
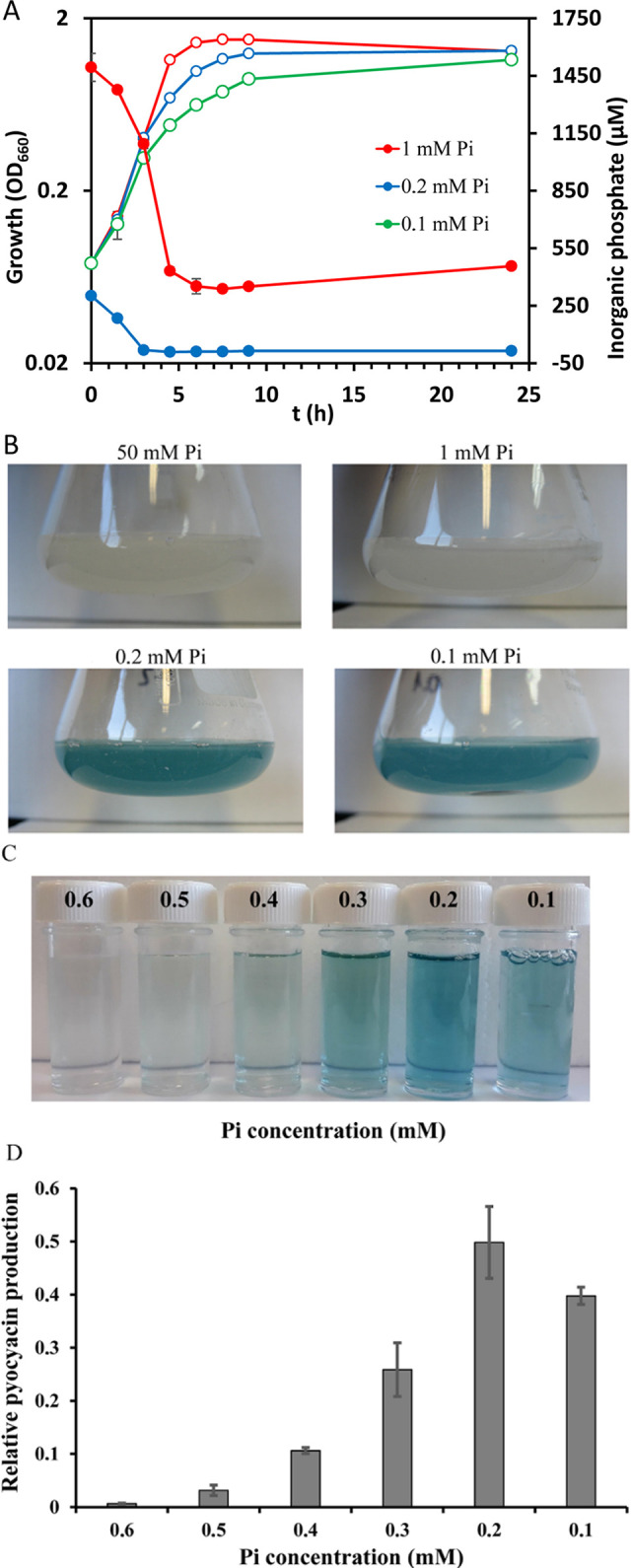
Growth of P. aeruginosa in the presence of different inorganic phosphate (Pi) concentrations. (A) Growth curves in minimal medium supplemented with different concentrations of Pi. The growth kinetics of cultures grown in 50 mM and 1 mM Pi are almost identical; hence the curve for 50 mM Pi has been omitted. The following growth rates were derived (in number of generations per h): 50 mM Pi, 1.01 ± 0.03; 1 mM Pi, 1.06 ± 0.03; 0.2 mM Pi, 0.87 ± 0.02; 0.1 mM Pi, 0.71 ± 0.02. The Pi concentration in supernatants is also shown. Growth (open symbols), Pi consumption (filled symbols). (B) Erlenmeyer flasks containing PA cultures after culture for 24 h at 37°C. (C) Cell-free supernatants from PA cultures grown in the presence of different Pi concentrations. (D) Quantification of pyocyanin from supernatants shown in C. Data were normalized using the cell density.

To determine the effect of different Pi concentrations on the regulation of virulence in PA, initial experiments were conducted to study the production of the virulence factor pyocyanin as a proxy. The canonical reference strain in PA research, PAO1, was used as a model. We conducted growth experiments in minimal medium (MM) supplemented with different Pi concentrations. Growth in the presence of 50 and 1 mM could be considered identical, whereas a slight reduction in the growth rate was observed in the presence of 0.2 mM Pi (Fig. 1A). This deviation was significantly larger at 0.1 mM Pi (Fig. 1A). The visual inspection of the Erlenmeyer flasks at the end of the growth experiment (Fig. 1B) revealed an intense blue-green color at 0.2 mM and 0.1 mM, indicative of an elevated pyocyanin production, whereas the cultures with 1 and 50 mM were pale. To precisely determine the Pi concentration range at which pyocyanin production was altered, we repeated growth experiments incrementing the Pi concentration in 0.1 mM steps and extracted pyocyanin (Fig. 1C) for its spectrometric quantification (Fig. 1D). Experiments showed that the transition occurs between 0.6 mM and 0.2 mM Pi. This 3-fold reduction in the Pi concentration caused about a 75-fold increase in the pyocyanin production (Fig. 1D), indicative of a regulatory mechanism characterized by very strong positive cooperativity.

### Phosphate starvation stimulates exoenzyme production and antibacterial activity.

**FIG 2:**
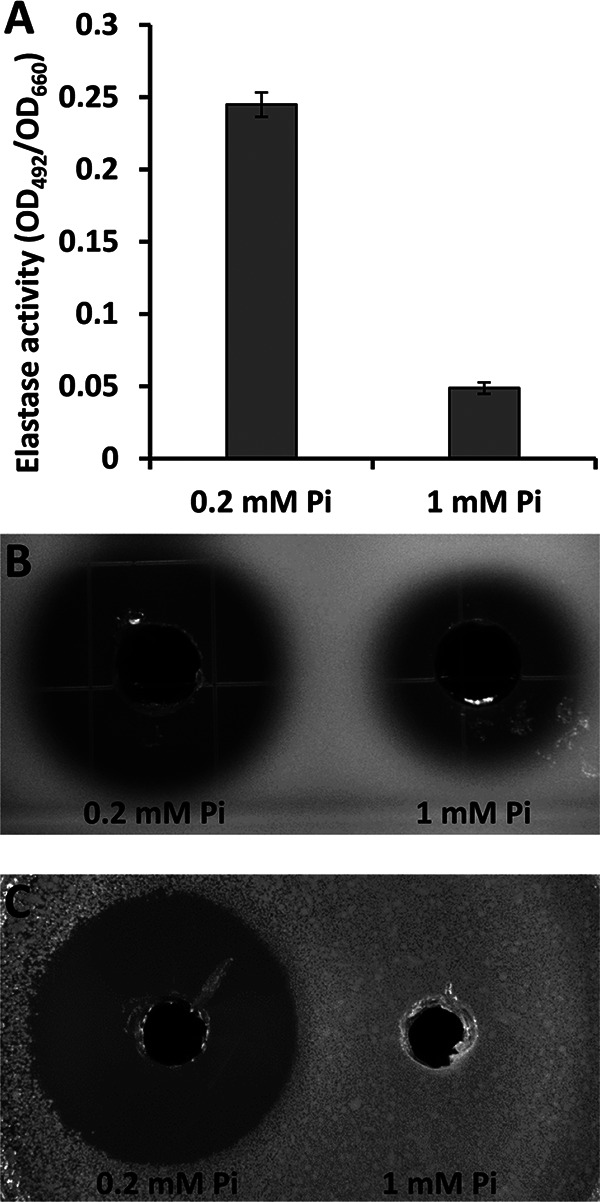
Exoenzyme production and antibacterial activity of P. aeruginosa PAO1 under Pi-deficient (0.2 mM) and -sufficient (1 mM) conditions. (A) Elastase activity in P. aeruginosa PAO1 supernatants. Data were normalized for culture density (OD_660_). Data are the means and standard deviations of three biological replicates. (B) Protease activity in P. aeruginosa PAO1 supernatants in skim milk agar plates. (C) Halos of antibiosis against Chromobacterium violaceum CV026 of filter-sterilized supernatants of P. aeruginosa PAO1. In B and C, the bioassays were conducted in triplicate and representative images are shown.

Among the hallmarks of PA virulence is the increased production of extracellular invasive enzymes such as elastases and proteases (47). As shown in Fig. 2A, the elastase activity of cell-free supernatants of PA grown in the presence of 0.2 mM Pi was about 5-fold higher than supernatants derived from cultures in 1 mM Pi. Fig. 2B illustrates the proteolytic capacity of these cell-free supernatants on skim milk agar, which is illustrated by the appearance of a clearance area around the bacterial colony. In analogy to the elastase activity, the protease activity under Pi starvation was significantly increased. Dramatic changes were observed in the measurements of the antibacterial activity of these supernatants, as monitored by a bioassay to kill Chromobacterium violaceum cells forming a lawn on agar plates. The placement of supernatants of PA grown with 0.2 mM Pi into holes punched into the plate caused a very significant antibacterial halo, whereas no antibacterial activity was detected for supernatants derived from cultures grown with 1 mM Pi (Fig. 2C).

### Reduction of Pi from 1 to 0.2 mM Pi causes large proteome changes.

**FIG 3:**
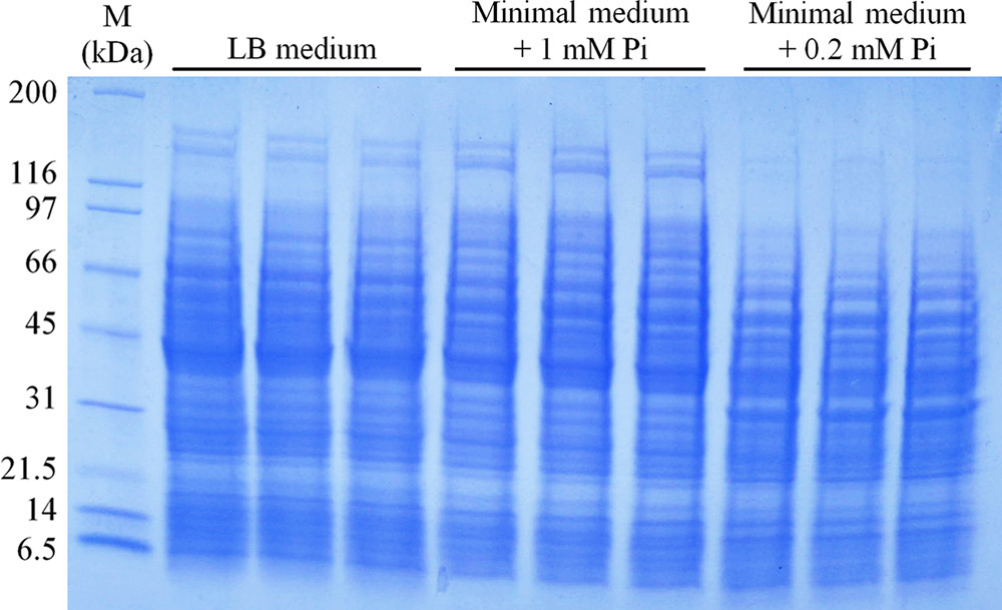
SDS-PAGE gel of protein extracts of P. aeruginosa cultures grown in LB rich medium or minimal medium supplemented with 1 mM or 0.2 mM Pi. Shown are the samples for three biological replicates that were used for the proteomics analysis. M, protein marker.

To get insight into the changes in the proteome caused by altered Pi levels, we prepared protein extracts from cultures grown in MM supplemented with 1 mM and 0.2 mM Pi as well as in LB rich medium. Samples were taken at an optical density at 660 nm (OD_660_) of 0.6, which implies that the supernatant Pi concentration at the moment of sample taking for cultures grown in 1 mM and 0.2 mM Pi was of approximately 700 and 13 μM, respectively (Fig. 1A). An SDS-PAGE gel of the three replicate samples used for proteomics is shown in Fig. 3. Whereas, visually the proteomes of PA grown in LB and MM + 1 mM Pi are similar, there are very important changes comparing samples grown in MM + 1 mM Pi and MM + 0.2 mM Pi (Fig. 3).

**FIG 4:**
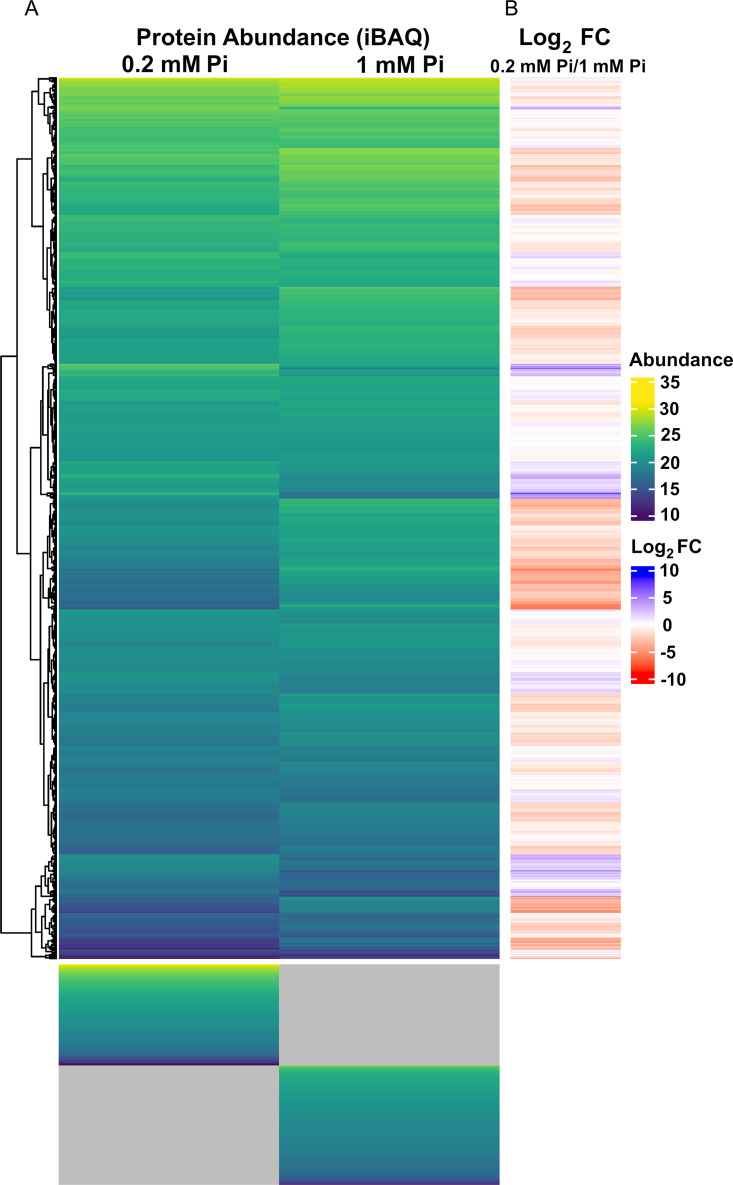
Heatmaps derived from P. aeruginosa growth in minimal medium supplemented with 0.2 mM or 1 mM Pi. (A) Protein abundance (intensity-based absolute quantification [iBAQ] values) in both samples. The gray shading indicates that the protein could not be detected in any of the three replicates of one condition, but was quantified in the three replicates of the other condition (referred to as “ON” or “OFF” at “low Pi” or “high Pi” in Data Set S2 and S3). (B) Log_2_ fold changes (FC) for growth in 0.2 mM compared to 1 mM Pi.

The proteomics analyses of these three conditions resulted in the quantification of a similar number of proteins, namely 2,716; 2,747; and 2,733 for the MM + 0.2 mM Pi, MM + 1 mM Pi, and LB samples, respectively (see Data Set S1 in the supplemental material). Considering that there are 5,587 annotated protein coding genes in the PA PAO1 genome (48), this corresponds to a coverage of about 49%. In this article, we analyze the changes in the proteome that occurred in cultures grown in the presence of 0.2 mM Pi compared to 1 mM Pi. Differentially abundant proteins are defined by a magnitude of the log_2_ fold change superior to 0.8 as well as a *P* value inferior to 0.01 in a Student's *t* test comparing the two growth conditions. In total, 295 and 1,022 proteins were found to be enriched or depleted, respectively, comparing growth in 0.2 mM Pi to 1 mM Pi. In addition, there were several cases where a given protein was detected in the three replicates of one growth condition but was not identified in the three replicates from the other growth conditions. It is very likely that the failure to detect a given protein in one growth condition is due to its concentration being below the dynamic detection range of the mass spectrometer. These proteins were thus included into the lists of depleted (Data Set S2) and accumulated proteins (Data Set S3). Growth in 0.2 mM Pi compared to 1 mM Pi resulted primarily in the downregulation of protein abundance (1,100 proteins [Data Set S2]), and in an accumulation of 371 proteins (Data Set S3) (Table 1, Fig. 4).

**TABLE 1:** Proteins with altered abundance in P. aeruginosa grown in minimal medium plus 0.2 mM Pi compared to minimal medium plus 1 mM Pi

Sense of change	Detected in both conditions and statistically different	Not detectable in the 0.2 mM Pi samples, but detected in the 1 mM Pi samples	Detected in the 0.2 mM Pi samples, but not detected in the 1 mM Pi samples	Total
Enriched proteins	295		76	371
Depleted proteins	1,022	78		1,100

### Lower abundance of proteins involved in metabolism and biosynthesis during Pi limitation.

**FIG 5:**
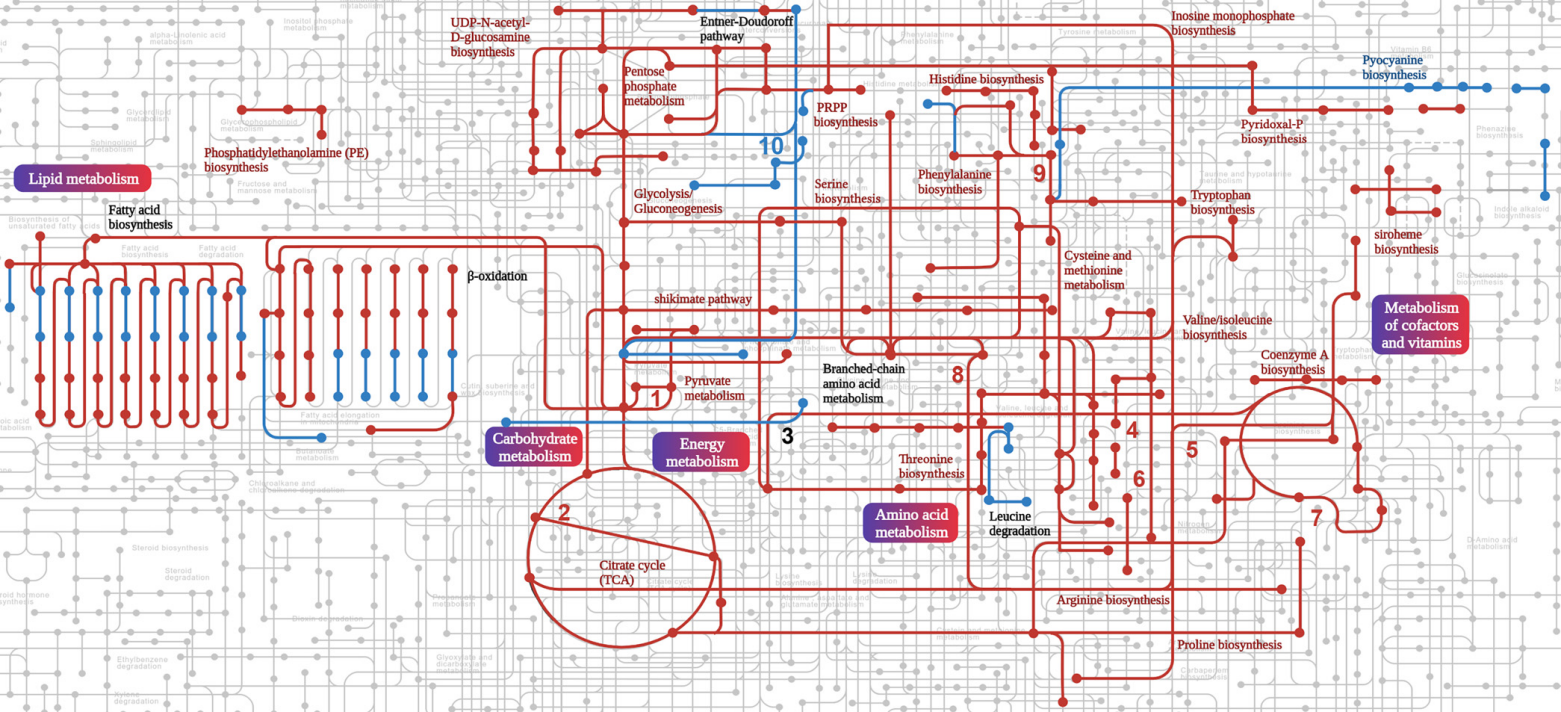
Metabolic map summarizing the principal differences in the proteomes of P. aeruginosa grown in minimal medium in the presence of 0.2 mM and 1 mM Pi. Up- and downregulated routes are shown in blue and red, respectively. Routes that contain both depleted and enriched proteins are annotated in black. 1, Phosphate acetyltransferase-acetate kinase pathway; 2, glyoxylate cycle; 3, lysine degradation; 4, isoleucine biosynthesis; 5, arginine and proline metabolism; 6, dissimilatory nitrate reduction; 7, polyamine biosynthesis; 8, glutathione biosynthesis; 9, tyrosine biosynthesis; 10, phosphonate and phosphinate metabolism.

**FIG 6:**
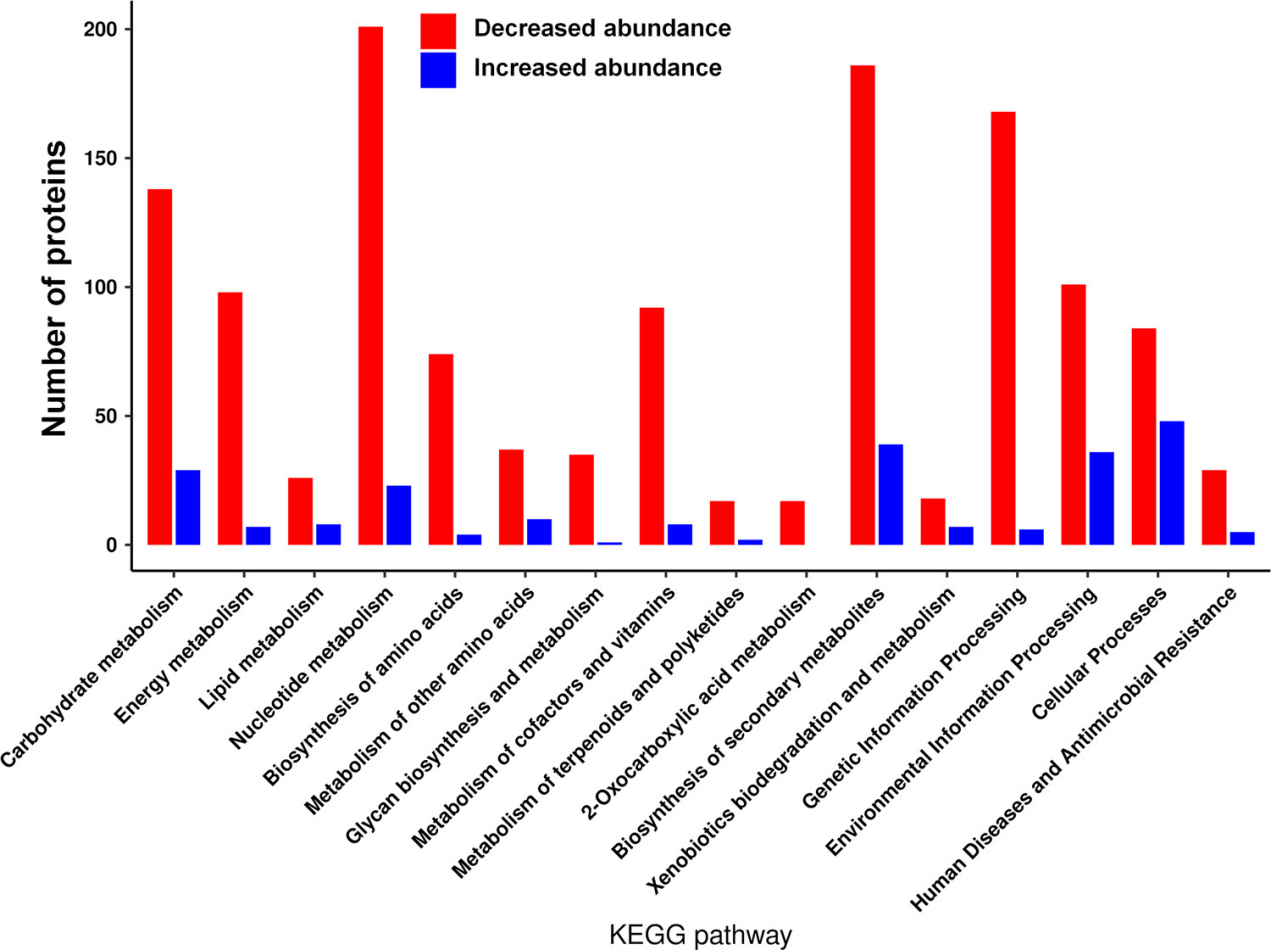
Analysis of differences in the proteomes of P. aeruginosa grown in the presence of 0.2 mM Pi compared to 1 mM. Classification of observed changes according to KEGG Orthology (KO) terms.

**FIG 7:**
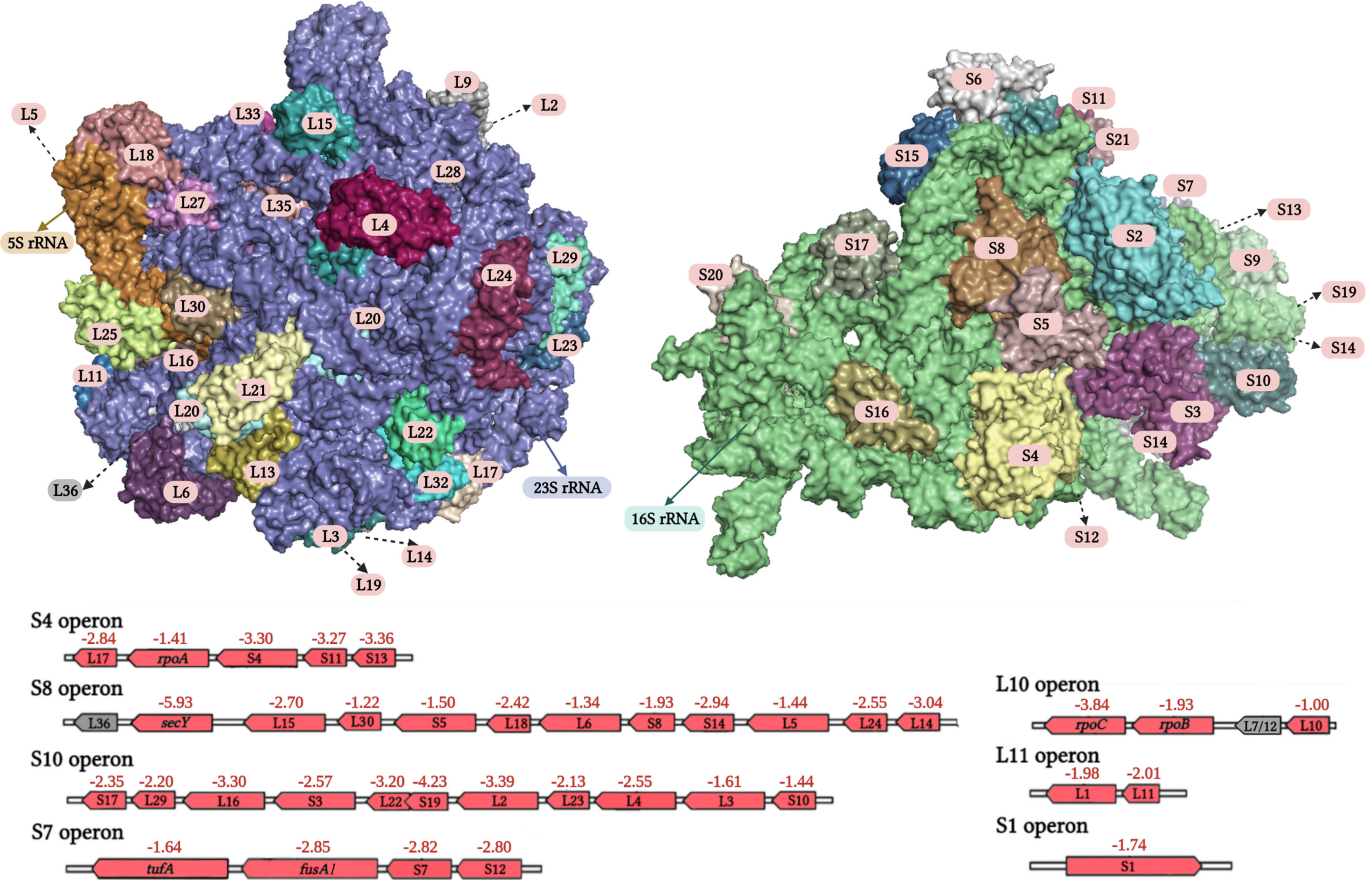
Changes in the abundance of ribosomal proteins of P. aeruginosa grown in the presence of 0.2 mM Pi compared to 1 mM. Red, decrease; gray, no significant difference. The numbers indicate the log_2_ fold change of iBAQ values. The fact that the fold change values are associated with genes does not imply that regulation is at the transcriptional level. The upper part shows the three-dimensional structures of the large and small ribosomal subunits of the PA ribosome (pdb: 6SPD).

The reduction in the Pi concentration resulted in lowered amounts of about 40% of the detected proteins that correspond to ~20% of the proteins encoded in the genome of PAO1 (Data Set S2). The 10 proteins with the strongest depletion are listed in Table 2 and include proteins involved in the general metabolism, like the maintenance of redox homeostasis or respiratory electron transfer chain, transport, biosynthesis, and catabolism. Fig. 5 shows a map of PA metabolic processes in which the downregulated processes are highlighted in red and the classification of differentially abundant proteins according to the KEGG Orthology (KO terms) is shown in Fig. 6. Major downregulated processes include the glycolysis/gluconeogenesis and Krebs cycle, the glyoxylate cycle, the pentose phosphate metabolism, and the pathway for dissimilatory nitrate reduction. Importantly, biosynthetic pathways for many amino acids, phosphatidylethanolamine, inosine 5’-monophosphate, pyridoxal phosphate, Coenzyme A, UDP-*N*-acetyl-d-glucosamine, siroheme, polyamines, and glutathione were also downregulated (Fig. 5 and 6; Data Set S2). Furthermore, protein levels for several metabolic pathways were reduced, like those for different amino biosynthesis, and phosphonate and phosphinate metabolism. Several enzymes of the fatty acid biosynthesis and degradation as well as the Entner-Doudoroff pathways were depleted whereas others showed increased levels (Fig. 5 and 6). The reduction in the Pi level caused significantly lowered amounts of enzymes in the biosynthesis and metabolism of all major groups of biological molecules, like carbohydrates, lipids, amino acids, cofactors, and vitamins as well as secondary metabolites (Fig. 6). Next to the reduction in the abundance of proteins involved in the biosynthesis and metabolism of biomolecules, there were numerous proteins with decreased amounts that participate in energy metabolism as well as signal processing like transcriptional regulation. The fact that the primary consequence of the reduction in Pi from 1 mM to 0.2 mM is a reduction in abundance of 1,100 proteins is also reflected in a general depletion of ribosomal proteins. In total, 36 ribosomal proteins were detected at lower levels with an average magnitude of 2.51 ± 0.98 log_2_ fold change that corresponds to about a 6-fold mean reduction of ribosomal proteins under Pi-limiting conditions (Fig. 7).

**TABLE 2:** Proteins with largest changes in abundance after growth in minimal medium plus 0.2 mM Pi compared to minimal medium plus 1 mM Pi

Locus tag	Gene	Protein name	Protein function/comment	Log_2_ FC	Ref.
Depleted					
PA0196	*pntB*	Pyridine nucleotide transhydrogenase, beta subunit	Regulation of NAD(P)^+^/NAD(P)H redox homeostasis	−7.27	111
PA1680		Predicted 3-oxoadipate enol-lactonase activity	Predicted role in benzoate catabolism	−6.46	112
PA1555	*ccoP2*	Cytochrome c oxidase, cbb3-type, CcoP subunit	Respiratory electron transfer chain component	−6.36	
PA2840		ATP-dependent RNA helicase	Regulation of type 3 secretion system	−6.30	113
PA1297	*aitP*	Metal transporter	Fe^2+^ and Co^2+^ export	−6.18	114
PA3321		Transcriptional regulator		−6.10	
PA0141		Polyphosphate kinase	GTP synthesis	−6.06	115
PA5118	*thiI*	Thiazole biosynthesis protein	Thiazole synthesis	−6.02	
PA1964		ATP-binding component of ABC transporter	Transport	−5.98	
PA4243	*secY*	Secretion protein SecY	Protein export	−5.93	
Enriched					
PA3330		Probable short-chain dehydrogenase	Regulated by quorum sensing	8.62	116
PA4214^*a*^ PA1903^*a*^	*phzE_1_* *phzE_2_*	Phenazine biosynthesis protein PhzE Phenazine biosynthesis protein PhzE	Phenazine (pyocyanin) biosynthesis, host interaction Phenazine (pyocyanin) biosynthesis, host interaction	7.90 7.90	117
PA4216^*a*^ PA1905^*a*^	*phzG_1_* *phzG_2_*	Probable pyrridoxamine 5′-phosphate oxidase Probable pyrridoxamine 59-phosphate oxidase		7.33 7.33	
PA3280	*oprO*	Pyrophosphate-specific outer membrane porin OprO	Phosphate and diphosphate transport	6.45	51
PA3279	*oprP*	Phosphate-specific outer membrane porin OprP		6.36	
PA5360	*phoB*	Two-component response regulator PhoB	Transcriptional regulation in response to Pi starvation	6.33	118
PA4211	*phzB_1_*	Phenazine biosynthesis protein	Phenazine biosynthesis	6.13	117
PA0690	*pdtA*	Phosphate depletion regulated two-partner secretion system partner A	Secretion system involved in virulence	5.97	119
PA0696		Hypothetical protein	Unknown, part of the σ^VreI^ regulon, upregulated by Pi	5.92	120, 121
PA0698		Hypothetical protein		5.80	

a Proteins of the same sequence.

### Many accumulated proteins under Pi-limiting conditions participate in virulence processes.

Among the proteins with the highest enrichment under Pi limitation (Table 2) were 5 proteins required for the synthesis of phenazine virulence factors (e.g., pyocyanin), 3 proteins involved in phosphate transport (OprO, OprP) and regulation (PhoB), a protein of a secretion system (PdtA), and two proteins of unknown function that belong to the regulon of the VreRI extracytoplasmic function (ECF) sigma factor system that is active during infection, promoting transcription of potential virulence determinants (49). An analysis of the totality of accumulated proteins permits their classification into several categories (Table 3) that are mainly related to phosphate acquisition or virulence processes.

**TABLE 3:** A selection of proteins with increased abundance comparing growth in minimal medium plus 0.2 mM Pi with minimal medium plus 1 mM Pi

Locus tag	Gene	Protein name/function	Log_2_ FC^*a*^
Proteins related to phosphate and phosphonate transport and regulation			
PA3279	*oprP*	Phosphate-specific outer membrane porin OprP precursor	6.36
PA3280	*oprO*	Pyrophosphate-specific outer membrane porin OprO precursor	6.45
PA3383	*phnD*	Binding protein component of ABC phosphonate transporter	3.23
PA3384	*phnC*	ATP-binding component of ABC phosphonate transporter	ON/OFF
PA5360	*phoB*	Two-component response regulator PhoB	6.33
PA5365	*phoU*	Phosphate uptake regulatory protein PhoU	3.56
PA5366	*pstB*	ATP-binding component of ABC phosphate transporter	3.44
PA5367	*pstA*	Membrane protein component of ABC phosphate transporter	3.73
PA5368	*pstC*	Membrane protein component of ABC phosphate transporter	2.68
PA5369	*pstS*	Phosphate ABC transporter, periplasmic phosphate-binding protein	2.66
Secretion systems			
Type II secretion systems			
PA0681	*hxcT*	Hxc type II secretion system	ON/OFF
PA0684	*hxcZ*	Hxc type II secretion system	ON/OFF
PA0685	*hxcQ*	Hxc type II secretion system	ON/OFF
PA0688	*lapA*	Alkaline phosphatase A (secreted by Hxc secretion system)	ON/OFF
PA0689	*lapB*	Alkaline phosphatase B (secreted by Hxc secretion system)	ON/OFF
PA0690	*pdtA*	Phosphate depletion regulated TPS partner A, type II secretion system	ON/OFF
PA3105	*xcpQ*	Xcp type II secretion system	2.67
PA3102	*xcpS*	Xcp type II secretion system	ON/OFF
PA0026	*plcB*	Phospholipase C (secreted by Xcp secretion system)	ON/OFF
PA0852	*cbpD*	Chitin-binding protein CbpD (secreted by Xcp secretion system)	4.46
PA3296	*phoA*	Alkaline phosphatase (secreted by Xcp secretion system)	3.03
PA3319	*plcN*	Nonhemolytic phospholipase C (secreted by Xcp secretion system)	ON/OFF
PA2939	*paAP*	Aminopeptidase (secreted by Xcp secretion system)	ON/OFF
PA0572	*pmpA*	Putative protease (secreted by Xcp secretion system)	5.10
PA1871	*lasA*	Elastase A (secreted by Xcp secretion system)	ON/OFF
PA0347	*glpQ*	Glycerophosphoryl diester phosphodiesterase (secreted by Xcp secretion system)	5.31
PA3910	*eddA*	Extracelullar DNA degradation protein (secreted by Xcp secretion system)	ON/OFF
Type VI secretion systems			
PA0082	*tssA1*	Type VI secretion system H1	1.22
PA0083	*tssB1*	Type VI secretion system H1	0.85
PA0086	*tagJ1*	Type VI secretion system H1	0.82
PA2363	*hsiJ3*	Type VI secretion system H3	ON/OFF
PA2365	*hsiB3*	Type VI secretion system H3	ON/OFF
PA2366	*hsiC3*	Type VI secretion system H3	ON/OFF
PA3905	*tecT*	Type VI effector chaperone for Tox-Release	1.46
PA0263^*b*^ PA5267^*b*^ PA1512^*b*^	*hcpC* *hcpB* *hcpA*	Type VI secretion system (protein of the spear) Type VI secretion system (protein of the spear) Type VI secretion system (protein of the spear)	2.35 2.35 2.35
PA1844	*tse1*	Type VI secretion system (secreted protein, peptidoglycan amidase)	1.30
Other enzymes involved in Pi metabolism (phosphatases, phospholipases, and diesterases)			
PA3885	*tpbA*	Protein tyrosine phosphatase	4.02
PA5292	*pchP*	Phosphorylcholine phosphatase	1.82
PA0843	*plcR*	Phospholipase accessory protein PlcR precursor	ON/OFF
PA2856	*tesA*	Lysophospholipase A	1.1
PA2990		Probable phosphodiesterase	0.95
Pyocyanin synthesis and activity			
PA4209	*phzM*	Phenazine-specific methyltransferase	2.99
PA4210	*phzA_1_*	Phenazine biosynthesis protein	ON/OFF
PA4211 PA1900	*phzB_1_* *phzB_2_*	Phenazine biosynthesis protein Phenazine synthesis protein	6.13 4.16
PA4213^*b*^ PA1902^*b*^	*phzD_1_* *phzD_2_*	Phenazine biosynthesis proteins Phenazine biosynthesis proteins	ON/OFF ON/OFF
PA4214^*b*^ PA1903^*b*^	*phzE_1_* *phzE_2_*	Phenazine biosynthesis proteins Phenazine biosynthesis proteins	7.9 7.9
PA4215^*b*^ PA1904^*b*^	*phzF_1_* *phzF_2_*	Phenazine biosynthesis proteins Phenazine biosynthesis proteins	4.46 4.46
PA4216^*b*^ PA1905^*b*^	*phzG_1_* *phzG_2_*	Pyridoxamine 5′-phosphate oxidase Pyridoxamine 59-phosphate oxidase	7.33 7.33
PA4217	*phzS*	Flavin-containing monooxygenase	2.92
Che_2_ chemosensory pathway			
PA0176	*aer_2_*	Chemoreceptor Aer_2_/McpB	1.62
PA0177	*cheW_2_*	Coupling protein	3.33
PA0179	*cheY_2_*	Response regulator	2.53
Quorum sensing			
PA0997	*pqsB*	HHQ/PQS synthesis	3.74
PA0998	*pqsC*	HHQ/PQS synthesis	2.59
PA0999	*pqsD*	3-oxoacyl-[acyl-carrier-protein] synthase III	4.43
PA1001	*phnA*	Anthranilate synthase component I	ON/OFF
PA1002	*phnB*	Anthranilate synthase component II	ON/OFF
PA2303	*ambD*	Synthesis of the IQS signal	3.63
PA2304	*ambC*	Synthesis of the IQS signal	3.37
PA3476	*rhlI*	C4-HSL autoinducer synthesis protein	2.22
PA3477	*rhlR*	Transcriptional regulator RhlR	3.88
Signal transduction			
PA0675	*vreI*	ECF sigma factor	ON/OFF
PA0676	*vreR*	Sigma factor regulator	ON/OFF
PA0696		Unknown function, part of the σ^VreI^ regulon	5.92
PA0698		Unknown function, part of the σ^VreI^ regulon	5.80
PA2047	*cmrA*	Transcriptional regulator of multidrug efflux pump	1.66
PA2895	*sbrR*	Anti-sigma factor, inhibits SbrI	1.43
PA2899	*atvR*	Atypical virulence-related response regulator AtvR	1.52
PA3161	*himD*	Integration host factor beta subunit	1.67
PA3347	*hsbA*	HptB-dependent secretion and biofilm anti-sigma factor	1.41
PA3385	*amrZ*	Alginate and motility regulator Z	1.81
PA3622	*rpoS*	Sigma factor RpoS	2.33
PA3678	*mexL*	Transcriptional repressor of the *mexJK* multidrug efflux operon	0.81
PA4101	*bfmR*	Response regulator involved in biofilm formation	2.17
PA4381	*colR*	Response regulator involved in polymyxin resistance	1.36
PA4608	*mapZ*	C-di-GMP-binding adaptor protein, interacts with a chemotaxis methyltransferase to control flagellar motor switching	1.52
PA4778	*cueR*	Transcriptional regulator activated by LasR	0.81
PA4878	*brlR*	Pyocyanin binding transcriptional regulator that activates multidrug efflux pump	3.14
PA5261	*algR*	Alginate biosynthesis regulator	1.12
PA5360	*phoB*	Two-component response regulator PhoB	6.33
Other virulence related genes			
PA0122	*rahU*	Rhamnolipid-interacting protein that modulates innate immunity and inflammation in host cells. Involved in biofilm formation	0.91
PA0355	*pfpi*	Protease involved in antibiotic resistance, swarming and biofilm formation	4.05
PA1249	*aprA*	Alkaline protease, degrades transferrin, complement proteins, cytokines, and components of the extracellular matrix	ON/OFF
PA3479	*rhlA*	Rhamnosyltransferase chain A	ON/OFF
PA3550	*algF*	Alginate o-acetylltransferase	2.53
PA3552	*arnB*	Uridine 5′-(beta-1-threo-pentapyranosyl-4-ulose diphosphate) aminotransferase with critical role in colistin resistance	ON/OFF
PA3692	*lptF*	Lipotoxin F, outer membrane protein, necessary for adhesion to human cells	1.34
PA4379	*warA*	Methyltransfrase involved in LPS biosynthesis	ON/OFF
PA4667	*lbcA*	Lipoprotein binding partner of CtpA, required for type III secretion system function and virulence	0.91

a ON/OFF corresponds to proteins that were detected in the three replicates of samples derived from growth in 0.2 mM Pi, but that were not detected any of the three replicates of samples derived from growth at 1 mM Pi.

b Proteins of the same sequence.

**(i) Proteins related to phosphate and phosphonate transport and regulation.** As mentioned above, the induction of pyocyanin production is a highly cooperative process (Fig. 1B–D). Pi is sensed by a complex comprising the PstABCS high-affinity phosphate transporter, the PhoU coupling protein and the PhoRB two-component system (50). The reduction in Pi in the growth medium resulted in a higher abundance of these proteins involved in Pi transport and sensing (Table 3; Data Set S3). OprP and OprO are outer membrane channels that permit the specific uptake of phosphate or pyrophosphate (51, 52) and under Pi starvation both proteins were found to be enriched at similar levels, namely, by factors of 82- and 87-fold (Table 3), indicating that these vicinal genes are subject to similar regulatory processes. Transcript levels of *oprO* and *oprP* were found to be upregulated when PA makes contact with lung epithelial cells (53). Next to the higher abundance of the PstABCS phosphate transporter, two of the three subunits of the PhnCDE phosphonate transporter were also detected in larger amounts. Phosphonates are phosphorus-containing organic molecules in which the phosphorous atom is linked directly to a carbon atom in a stable chemical bond. However, studies of E. coli PhnCDE revealed that it also transports Pi and the authors conclude that PhnCDE has to be considered a genuine phosphate transporter (54).

**FIG 8:**
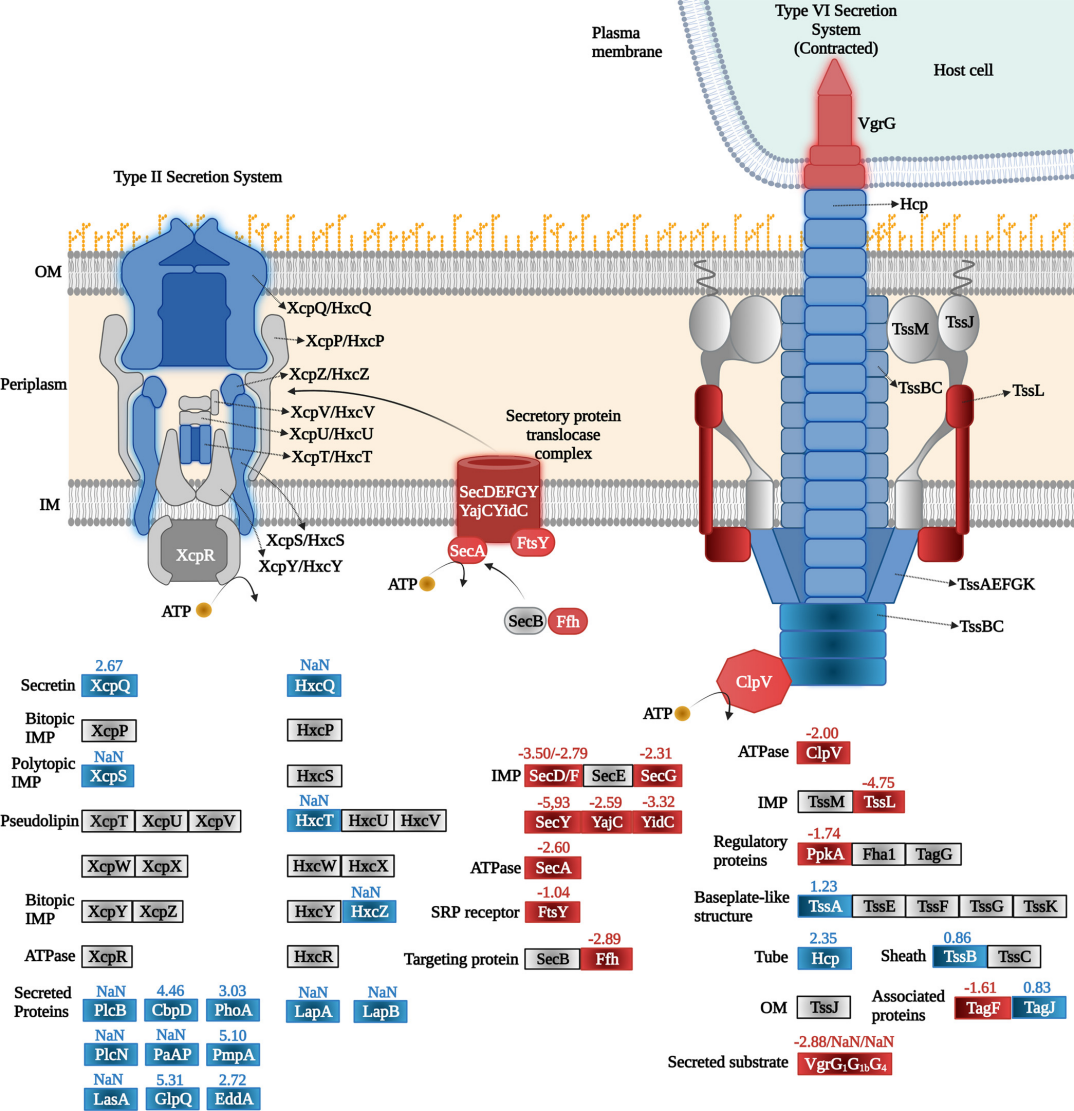
Changes in the abundance of secretion system proteins of P. aeruginosa grown in the presence of 0.2 mM Pi compared to 1 mM. Blue, increase; red, decrease; gray, no significant difference; NaN, not a number (not detected in the three replicates of the 1 mM Pi samples but identified in the three replicates of the 0.2 mM Pi sample). The numbers indicate the log_2_ fold changes of iBAQ values.

**(ii) Secretion systems.** Protein secretion systems are multiprotein complexes that are used to release proteins, toxins, or enzymes, for example, enzymes that hydrolyze complex carbon sources or proteins that capture essential elements such as iron (55). These systems are also used to colonize and survive within eukaryotic hosts, causing acute or chronic infections, subverting the host cell response, and escaping the immune system (56). Pi limitation modulated protein levels of both type II and type VI secretion systems (Fig. 8, Table 3).

PA has two homologous type II secretion systems, Hxc and Xcp, and several proteins of both systems were among the enriched proteins under Pi limitation (Fig. 8, Table 3). This accumulation was accompanied by an increase in the levels of numerous proteins that were shown to be secreted by these systems (Fig. 8). Some of these proteins, namely, different phosphatases and phosphodiesterases, are involved in the Pi metabolism, whereas others were proteases and a chitin-binding protein (Table 3). Among the accumulated proteins that are secreted by the type II system is the protease LasA that is among the most relevant PA virulence factors. LasA acts synergistically with elastase LasB to degrade elastin (57). Elastin fibers are abundant in different human tissues like lung, skin, or auricular cartilage (58). The importance of LasA and LasB as virulence factors is demonstrated by a study reporting an about 70% reduction in *in vitro* invasion of epithelia cells for mutants deficient in one of the enzymes, whereas loss of both enzymes caused a further decrease in the invasive capacity of the cell (59). As indicated above, we observed an increase of about 5-fold in elastase levels *in vitro* at 0.2 mM Pi with respect to 1 mM Pi (Fig. 2A).

Two other exported proteins, the LapA and LapB phosphatases, were both not detected in the 1 mM Pi condition, whereas significant levels were detected under Pi scarceness (Data Set S1). In a study monitoring biofilm formation using an *ex vivo* chronic wound model, the *lapA* gene was the most upregulated (log_2_ FC = 11.32) compared to planktonic growth, indicating that this phosphatase plays a key role in wound infection, making it a potential drug target (60). LapA/LapB and PhoA, another phosphatase with increased abundance, are thought to be independently exported by the Hxc and Xcp type II secretion systems, respectively (61, 62). The secretion of LapA via the Hxc system is considered a complementary means to acquire Pi in case PhoA secretion by the Hcp system has become limited (61).

The phospholipases PlcB and PlcN are secreted by the Xcp type II secretion system and a hyperbiofilm phenotype was reported in mutants defective in *plcB* or *plcN* (63). Both proteins were not detected in 1 mM Pi but were present at significant amounts under Pi limitation. Another enriched protein, EddA, has alkaline phosphatase and phosphodiesterase activities and was found to be essential for the defense against extracellular traps that are produced by neutrophil immune cells as a mechanism to fight microbial pathogens (64).

In PA, there are three gene clusters that encode three homologous type VI secretion systems (H1- to H3-T6SS) (65). These systems are molecular nanomachines that inject effectors into target eukaryotic and prokaryotic cells and promote survival under harmful conditions, challenging survival of competitor organisms and helping PA to prevail in specific environments (66). Pi starvation had a differential impact on the levels of type VI secretion systems (Table 3, Fig. 8). Several proteins encoded in the H3 cluster were accumulated (Table 3), whereas the level of proteins encoded in cluster H2 remained unchanged. Pi starvation had a differential impact on proteins of the H1 cluster for which abundances were either increased or decreased (Table 3, Fig. 8; Data Set S2). One of the toxins secreted by the H1 system, Tse1, is among the accumulated proteins under Pi limitation.

**(iii) Other enzymes involved in Pi metabolism.** Next to the enriched phosphatases and phospholipases that are substrates for secretion systems, several other enzymes involved in Pi metabolism showed increased levels in Pi-limited conditions (Table 3). Among them is the tyrosine phosphatase TpbA that plays a key role in PA physiology. TpbA specifically controls the phosphorylation state of the transmembrane diguanylate cyclase TpbB (PA1120) by dephosphorylating Tyr and Ser/Thr residues. This control of the phosphorylation state regulates TpbB activity which in turn determines central features in PA physiology like the rugose colony morphology, biofilm production, and swarming motility (67, 68). Pi starvation also increased levels of the phosphorylcholine phosphatase PchP. Among the mechanisms required for the colonization of different tissues is the breakdown of host membrane phospholipids, a reaction catalyzed by PchP resulting in choline and Pi (69). A mutant in *plcR* was 200-fold less virulent in a mouse infection model (70). PlcR is an accessory protein that was found to be essential for the secretion of the PlcH phospholipase. In our study, PlcR was enriched (Table 3) whereas PlcH was depleted (log_2_ FC = 3; Data Set S2) (71). It may be possible that PlcR is also required for the secretion of other phospholipases.

**FIG 9:**
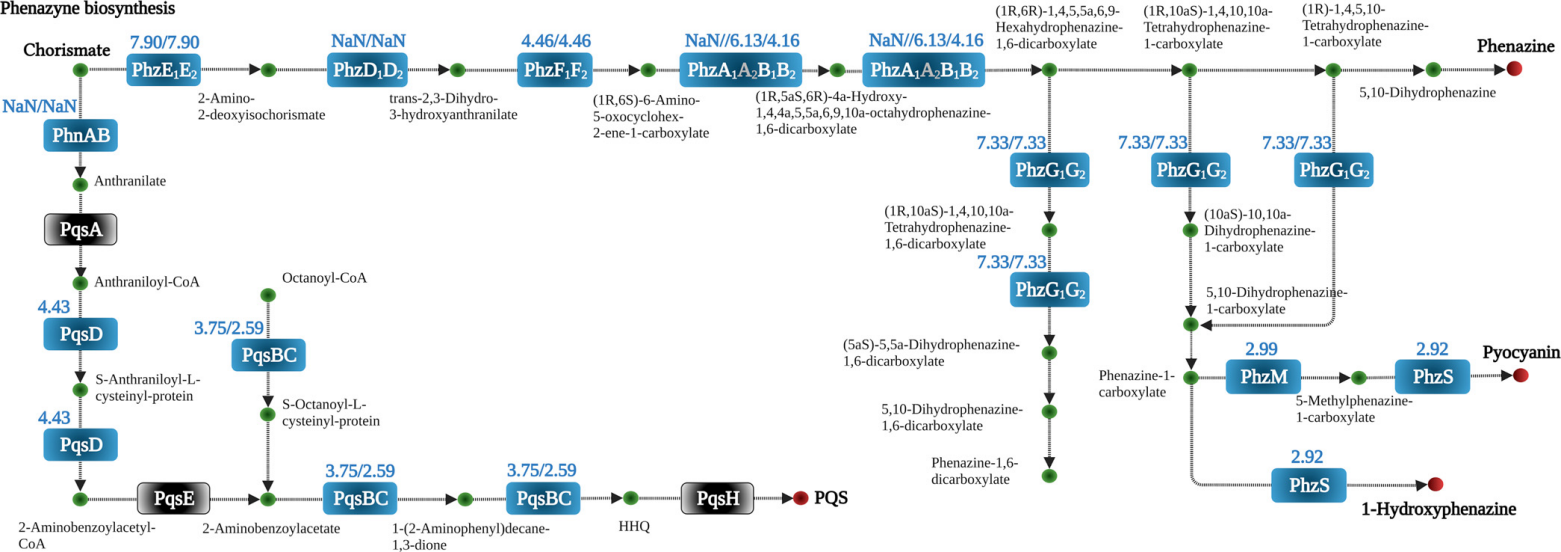
Changes in the abundance of proteins involved in the synthesis of quorum sensing signals and phenazines of P. aeruginosa grown in the presence of 0.2 mM Pi compared to 1 mM. Blue, increase; gray, no significant difference; NaN, not a number (not detected in the three replicates of the 1 mM Pi sample but identified in the three replicates of the 0.2 mM Pi sample). The numbers indicate the log_2_ fold changes of iBAQ values. Values separated by a slash are those of the individual subunits of an enzyme.

**(iv) Pyocyanin synthesis and activity.** Chorismate is the end product of the shikimate pathway that was downregulated under Pi-limiting conditions (Fig. 5). However, the routes that convert chorismate into the QS signal 4-hydroxy-2-heptylquinoline (HHQ) and the phenazines pyocyanin, phenazine, and 1-hydroxyphenazine were strongly upregulated (Fig. 9). Several proteins involved in phenazine biosynthesis were among those with the largest increase, as detailed in Table 2, and pyocyanin production was dramatically enhanced under Pi starvation (Fig. 1B–D). Pyocyanin is a redox-active compound that is secreted into the medium. It is one of PA’s primary virulence factors and is required to establish infection (72). Due to its redox activity, pyocyanin toxicity has been associated with the inhibition of aerobic respiration and the production of reactive oxygen species (73), and promoting virulence by interfering with numerous cellular functions in host cells (74). The different phenazines are considered biomarkers of PA infections (72).

**FIG 10:**
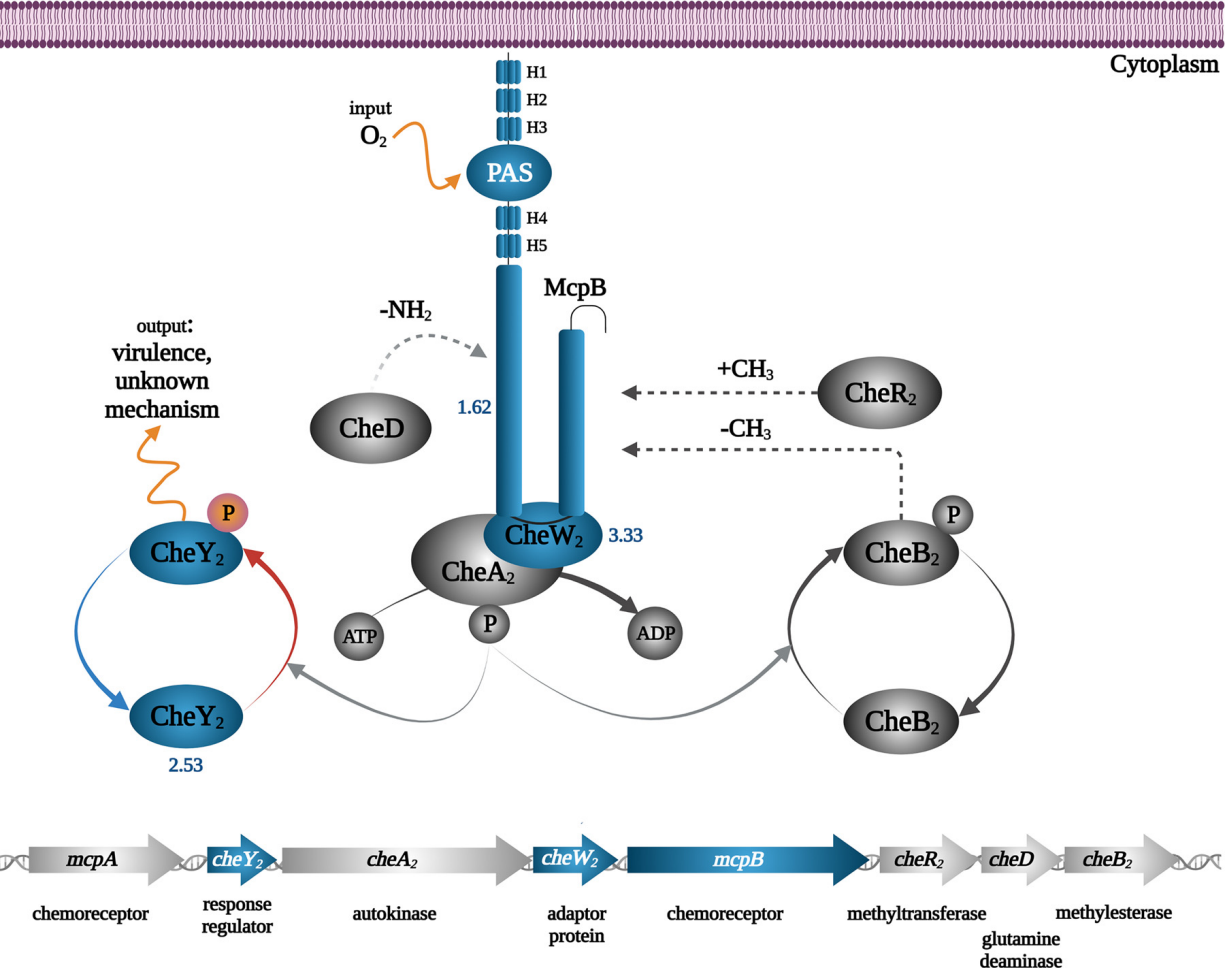
Changes in the abundance of signaling proteins of the Che_2_ pathway of P. aeruginosa grown in the presence of 0.2 mM Pi compared to 1 mM. Blue, increase; gray, no significant difference or not detected; the numbers indicate the log_2_ fold changes of iBAQ values. H1 to H5: HAMP (found in histidine kinases, adenylate cyclases, methyl accepting proteins) domains.

**(v) Che_2_ chemosensory pathway.** PA has four chemosensory pathways. Of these, the Che pathway mediates chemotaxis and the Wsp pathway controls c-di-GMP levels, whereas the Chp pathway is associated with mechanosensing and type IV pili-based motility (75). Although the function of the fourth pathway, Che_2_, is unknown, there are multiple pieces of evidence indicating that it plays an important role in virulence. It has been shown that the deletion of the genes encoding the sole chemoreceptor that stimulates this pathway, Aer_2_/McpB, as well as the CheB_2_ methylesterase caused a reduction in virulence (76, 77). Under Pi scarceness, three proteins of the Che_2_ pathway were accumulated, namely, the McpB chemoreceptor, the CheW_2_ adaptor protein, and the CheA_2_ autokinase (Fig. 10). In accordance with our results, different genes of the Che_2_ pathway were upregulated in a transcriptomic study at low Pi levels (18).

**FIG 11:**
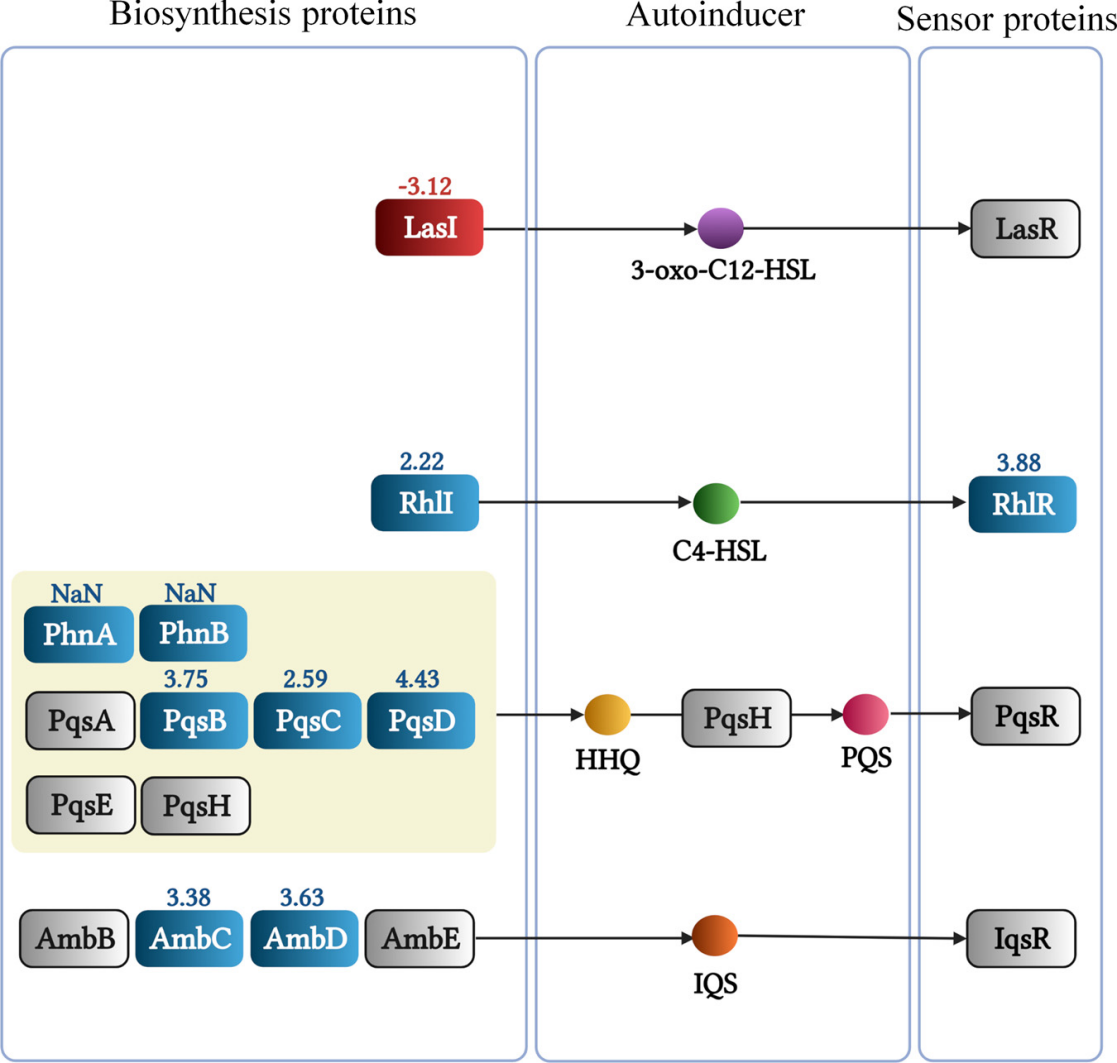
Changes in the abundance of proteins involved in quorum sensing of P. aeruginosa grown in the presence of 0.2 mM Pi compared to 1 mM. Blue, increase; red, decrease; gray, no significant difference or not detected; the numbers indicate the log_2_ fold changes of iBAQ values. NaN, not a number (not detected in the three replicates of the 1 mM Pi sample but identified in the three replicates of the 0.2 mM Pi sample). HSL, homoserine lactone; HHQ, 4-hydroxy-2-heptylquinoline; PQS, 2-heptyl-3-hydroxy-4-quinolone.

**(vi) Quorum sensing.** So far four QS systems have been reported in PA, namely, the Las, Rhl, Pqs, and Iqs systems. The Las and Rhl systems involve the synthesis and detection of the acylhomoserine lactone QS signals *N*-3-oxododecanoylhomoserine lactone (3-oxo-C12-HSL) and *N*-butanoylhomoserine lactone (C4-HSL), respectively (Fig. 11). Alternatively, the Pqs system uses HHQ and 2-heptyl-3-hydroxy-4-quinolone (PQS) as signals, whereas the Iqs system is based on the production of 2-(2-hydroxyphenyl)-thiazole-4-carbaldehyde (IQS) (Fig. 11) (78). Under Pi-sufficient conditions, the function of these four systems is closely interwoven with the Las system at the top of the regulatory hierarchy controlling the expression of different parts of the remaining three systems (78). The concerted action of these QS systems controls the expression of 10% of PA genes, including many virulence factor genes (73, 78). Many of the proteins known to be controlled by QS were detected at increased amounts in our study, including enzymes involved in the pyocyanin synthesis, the LasA protease, the PlcB phospholipase, Che_2_ pathway proteins, components of type II and type VI secretion system, or other proteins involved in virulence like the PfpI protease, lipotoxin F (LptF) or the RahU aegerolysin (Table 3) (78, 79). These data suggest a central role of the QS systems in mediating Pi responses. Pi starvation caused significant increases in the protein levels of the RhlI/RhlR pair and those of several proteins that are required for the synthesis of quinolone and IQS QS signals (Fig. 11). In contrast, an important depletion of the LasI synthase was noted, suggesting that the accumulation of the Rhl, Pqs, and Iqs proteins is not primarily mediated by the Las system under Pi-limited conditions, in accordance with transcriptomics studies (24, 80).

**(vii) Signal transduction proteins.** Pi scarceness also altered the levels of a significant number of signal transduction proteins of which the most relevant examples are listed in Table 3. Next to the very significant increase observed for proteins of the VreIR ECF system and the PhoB response regulator, notable increases were also observed for other response regulators, ColR, AtvR, and BfmR, as well as the transcription factors CmrA, MexL, and AmrZ. ColR is involved in conferring polymyxin resistance (81) and AtvR was associated with PA adaptation to anaerobic conditions during infection (82). BfmR was shown to be essential for biofilm maturation (83) and its activation caused a shift from acute to chronic infection (84). MexL, BrlR, and CmrA regulate the expression of multidrug efflux pumps (85, 86). Interestingly, pyocyanin was shown to bind to BrlR, in turn enhancing BrlR-DNA affinity (87). AmrZ regulates twitching motility and alginate biosynthesis, two processes required for biofilm development (88, 89). Furthermore, this regulator orchestrates the assembly of all three type VI secretion systems (90). Further proteins with increased levels include the RpoS sigma factor that plays a central role in QS-mediated regulation (91), and MapZ, that controls the methylation state of chemoreceptors and consequently chemotaxis (92), another important virulence treat (78).

**(viii) Other proteins involved in virulence.** Several additional proteins with diverse roles in virulence were among the accumulated proteins (Table 3). An example is the rhamnolipid-interacting protein RahU, that is under the control of the Rhl QS system (93). RahU is involved in biofilm formation (94) and was shown to interfere with host innate immunity by inhibiting the accumulation of nitric oxide and chemotaxis of different immune cells (95). The protease PfpI was upregulated more than 16-fold (Table 3) and mutants defective in *pfpI* showed an increased resistance to ciprofloxacin, reduced swarming motility, and biofilm formation (96). Higher protein amounts were also observed for the RhlA rhamnosyltransferase and the ArnB aminotransferase, two enzymes essential for the synthesis of rhamnolipids (97) and involved in the production of an arabinose derivative that forms part of lipopolysaccharides (LPSs) (98), respectively. Colistin is a last-resort antibiotic to fight PA and colistin resistant mutants showed increased amounts of ArnB (99). Furthermore, the WarA methyltransferase interacts with the LPS biogenesis machinery and contributes to the ability of PA to evade detection by the host (100), most likely due to the role of LPSs as host immune response modulator. LbcA is a lipoprotein that forms a complex with the CtpA protease. LbcA is essential for the proteolytic activity of CtpA that in turn is required for the function of a type III secretion system and virulence in a mouse model of acute pneumonia (101, 102). Lastly, it is worth mentioning that the outer membrane protein, LptF, which has been associated with antibiotic resistance (103), was also enriched (Table 3).

### Conclusions and future perspectives.

An increase in bacterial virulence caused by Pi limitation appears to be a general regulatory mechanism since it has been observed for several bacterial pathogens that differ in phylogeny, lifestyle, and virulence mechanisms (11–17). Phosphate therapy, or the reduction of bacterial virulence by the administration of Pi (or phosphate-containing compounds), is a promising approach to fight microbial pathogens that will not cause cytotoxicity nor the development of antibiotic resistance. The proof of concept for phosphate therapy has been obtained using primarily PA as a model organism (21, 29, 31, 32, 35, 36). In this study, we have determined the changes in the PA proteome caused by alterations in the Pi concentration.

Using the synthesis of pyocyanin as a proxy for the regulatory action of Pi, we show that changes occur in a very small window of Pi concentrations. Whereas almost no pyocyanin is synthesized in the presence of 0.6 mM Pi, a 3-fold decrease in the Pi concentration caused about a 75-fold increase in the pyocyanin levels, indicative of a highly cooperative process. Our proteomics analyses identified at least part of the molecular basis for the cooperativity observed. Pi is sensed by the PstABCS/PhoRBU complex (50), and Pi scarceness caused large increases in the protein levels of this sensory complex (Table 2 and 3) resulting in a feed-forward regulatory circuit and positive cooperativity.

**FIG 12:**
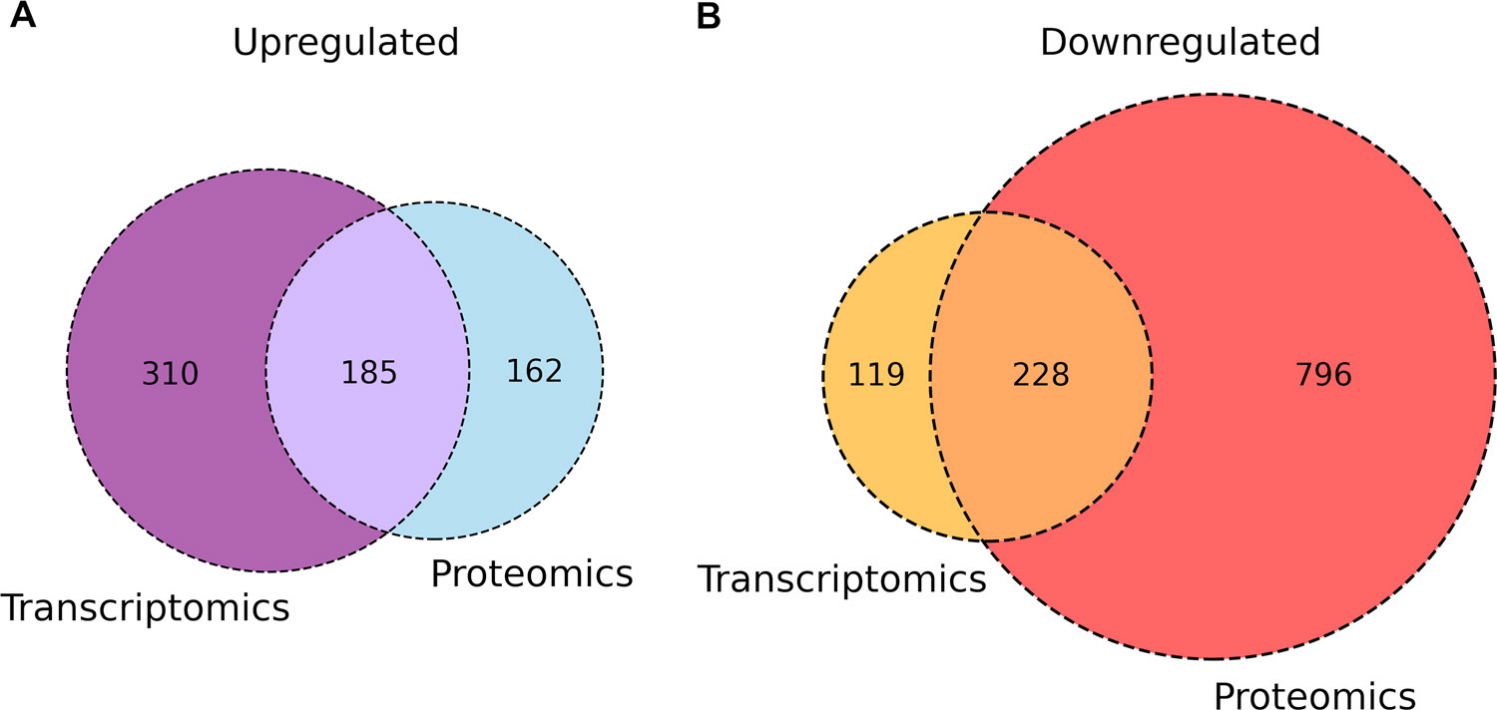
Venn diagram comparing genes/proteins regulated by an exposure of PA PAO1 to 0.2 mM and 1 mM Pi in a transcriptomics study (18) and our proteomics analysis. The culture conditions in both studies were identical. Since in the transcriptomics study, a cutoff 2-fold change was applied, the dysregulated proteins in the proteomics study were truncated at the same level.

As mentioned above, Pi scarceness increases the virulence of many different pathogens by an overexpression of different virulence factors. However, our study shows that of the 1,471 dysregulated proteins, the large majority, 1,100, were detected with lower abundance. Most of the depleted proteins are involved in general and energy metabolism, transport, and many different biosynthetic and catabolic routes (Fig. 5) or form part of the ribosome (Fig. 7). This issue is also illustrated by comparing the transcriptomics study of Bains et al. (18) with our proteomics data. In both studies, PAO1 was cultured under the same growth conditions and exposed to either 0.2 mM or 1 mM Pi. Fig. 12 is a Venn diagram of the up- and downregulated genes/proteins in both studies. Whereas a balance in the number of upregulated genes/proteins is observed between both studies, there is a large excess of downregulated proteins compared to the genes that were downregulated in the transcriptomics study (Fig. 12) (18). These data suggest the existence of strong posttranscriptional regulatory mechanisms that are responsible for the vast majority of depleted proteins.

Many of the accumulated proteins in our study are known to be regulated by quorum sensing, suggesting that QS is a key mechanism by which Pi induces the corresponding changes. In general, the four QS systems in PA are arranged in a hierarchical order with the Las system on top, controlling the activity of the Rhl, Pqs, and Iqs systems (78). However, this general scheme does not appear to apply to QS-mediated changes under Pi-scarce conditions. Indeed, a recent study (25) has shown that: (i) the Las system is globally dispensable for mediating responses to low Pi; (ii) LasR expression is not affected by phosphate limitation; (iii) PhoB directly regulates *rhlR* transcription; and (iv) mutation of *pqsR* reduced pyocyanin production as a whole, suggesting that under phosphate limitation, the Rhl system is on top of the QS regulatory hierarchy. However, contrary to these findings, an alternative study revealed that PhoB activated *lasI* expression, the gene encoding the homoserine lactone (HSL) synthesizing enzyme of the Las system, under phosphate depletion conditions (26). Our proteomics data largely support the notion that QS-mediated regulation in response to Pi limitation is Las independent and governed by the Rhl system. Pi scarceness significantly reduced LasI levels, did not alter LasR levels, but increased components of the Rhl (e.g., RhlI, RhlR, RhlA), Pqs (e.g., PhnA, PhnB, PqsB, PqsC, PqsD) and Iqs (e.g., AmbC, AmbD) systems (Fig. 9 and 11).

A recent review has classified the different virulence hallmarks of PA according to their function into the following 6 groups: factors for host colonization and bacterial motility, biofilm formation, production of destructive enzymes, toxic secondary metabolites, iron-chelating siderophores, and toxins (47). Representatives of all six categories were among the proteins that were upregulated by Pi limitation. Abundances of several key virulence factors identified for each of these six categories were increased in our study, including PstS, LasA, AprA, PlcB, PlcN, PhzABDEFG, RhlAIR, PqsBCD, and AlgFR (47). Pi limitation has been observed following surgical injury, leading to an increased bacterial virulence (20, 21). This enhanced virulence is due to an induction of proteins that belong to all six major categories of virulence hallmarks. However, the administration of Pi to patients to increase its concentration above 0.5 mM will result in a reversal of virulence protein induction. Phosphate therapy thus holds a strong promise as an alternative strategy to tackle microbial pathogens.

## MATERIALS AND METHODS

### Bacterial strains and growth conditions.

PA PAO1 was grown at 37°C with orbital shaking (200 rpm) in LB (5 g/L yeast extract, 10 g/L bactotryptone, 5 g/L NaCl) or MM (0.1 M HEPES, 7 mM [NH_4_]_2_SO_4_, 20 mM succinate, 1 mM MgSO_4_, 6 mg/L Fe-citrate, 300 mg/L HBO_3_, 50 mg/L ZnCl_2_, 30 mg/L MnCl_2_ × 4 H_2_O, 200 mg/L CoCl_2_, 10 mg/L CuCl_2_ × 2 H_2_O, 20 mg/L NiCl_2_ × 6 H_2_O, 30 mg/L NaMoO_4_ × 2 H_2_O, pH 7.0) containing 0.1 to 50 mM potassium phosphate (K_2_HPO_4_/KH_2_PO_4_, pH 7.0).

### Quantification of the supernatant Pi concentration during bacterial growth.

Determination of Pi concentration along the growth curve was carried out using the malachite green phosphate assay kit (Sigma-Aldrich; Catalog number MAK307) following the manufacturer’s instructions. Reaction assays were incubated for 30 min at room temperature for color development, followed by an absorbance measurement at 660 nm using a Sunrise plate reader from Tecan (Männedorf, Switzerland).

### Phenotypic assays.

Assays for pyocyanin quantification, antibacterial activity, and extracellular enzyme production (e.g., proteases and elastase) were carried out on culture supernatants from PA PAO1 cells grown in MM with different Pi concentrations. Overnight cultures of PAO1 grown in MM containing 1 mM inorganic phosphate (K_2_HPO_4_/KH_2_PO_4_) were washed twice with phosphate-free MM and then used to inoculate flasks containing 15 mL MM with 0.1 to 1 mM Pi at an initial OD_660_ of 0.075 and grown for 24 h at 37°C with orbital shaking (200 rpm). After overnight growth, bacterial cultures were centrifuged at 9,000 × *g* for 10 min and the supernatants were filtered (0.2 μm cutoff). The resulting filter-sterilized supernatants were used to conduct the following bioassays.

**(i) Pyocyanin quantification.** Pyocyanin was extracted into chloroform by mixing 7.5 mL of each of the supernatants with 4.5 mL of chloroform. Pyocyanin was further extracted from the solvent phase into 1.5 mL of 0.2 M HCl. The absorbance of the resulting solution was measured at 520 nm and data normalized against cell density at 660 nm.

**(ii) Antibacterial assays.** Antibiotic activity was tested by the agar lawn assay using 0.8% LB agar plates containing a top lawn of 200 μL of an overnight culture of Chromobacterium violaceum CV026 (104). Next, 300 μL of filter-sterilized PAO1 supernatants were added to wells punched into the plates and incubated at 30°C for 48 h.

**(iii) Elastase activity determination.** One milliliter supernatant was added to tubes containing 10 mg elastin-Congo red (ECR; Merck) and 1 mL of 0.1 M Tris/HCl, 1 mM CaCl_2_ (pH 7.0). Tubes were incubated at 37°C with shaking (200 rpm) for 24 h and the reactions stopped by the addition of 1 mL 0.7 M sodium phosphate buffer pH 6.0. Residual, solid ECR was removed by centrifugation and the OD_492_ of the reaction was measured. These measurements were normalized against the absorbance at OD_492_ of the cell-free supernatants prior to the addition of ECR and against cell density at 660 nm.

**Protease activity.** Qualitative proteolysis assays were conducted using the skim milk assay. Briefly, 300 μL supernatants were added to wells punched into the skim milk plates (1% skim milk – quarter-strength LB) and incubated at 30°C for 48 h. Skim milk proteolysis was evaluated as the zone of clearance from the well edge.

### Sample preparation for mass spectrometry (MS).

Overnight cultures of PA PAO1 grown in MM containing 1 mM Pi (K_2_HPO_4_/KH_2_PO_4_, pH 7.0) were washed twice with Pi-free MM. Washed cultures were then used to inoculate flasks with 50 mL MM containing high (1 mM) and low (0.2 mM) Pi concentrations at an OD_660_ of 0.075 and grown at 37°C with orbital shaking (200 rpm). At an OD_660_ of 0.6, 40 mL aliquots were centrifuged at 9,000 × *g* for 10 min. The resulting pellets were resuspended in 2 mL extraction buffer (6 M urea, 2 M thiourea, 50 mM Tris/CL, pH 7.5) and cells were broken by sonication. After centrifugation at 9,200 × *g* for 10 min, supernatants were collected, and protein concentration was determined by the Bradford assay (Bio-Rad, reference no. 500-0006). Supernatant aliquots were subsequently mixed with 5× Laemmli sample buffer containing 1 mM β-mercaptoethanol. After 1 h of incubation at room temperature, aliquots corresponding to 25 μg of protein were run on a 4 to 20% precast polyacrylamide gel (Bio-Rad, reference no. 4561094) at 95 V. The polyacrylamide gel was fixed with solution A (40% [vol/vol] methanol, 7% [vol/vol] acetic acid) for 10 min and stained with solution B (0.1% [wt/vol] Coomassie brilliant blue R [Sigma-Aldrich, Ref. B7920], 40% [vol/vol] methanol, 10% [vol/vol] acetic acid). The gel was finally destained with solution A. Proteins were prepared for tandem mass spectrometry analyses, as previously described (105). Briefly, gel lanes were fractionated into 10 gel pieces, cut into smaller blocks, and transferred into low binding tubes. Samples were washed until gel blocks were destained and dried in a vacuum centrifuge before they were covered with a trypsin solution. Samples were digested overnight at 37°C prior to peptide elution into water by ultrasonication treatment. The peptide-containing supernatants were transferred into fresh tubes, desiccated in a vacuum centrifuge and peptides were resolubilized in 0.1% (vol/vol) acetic acid for mass spectrometric analysis.

### MS/MS analysis.

LC-MS/MS analyses were performed on a LTQ Orbitrap VelosPro instrument (ThermoFisher Scientific, Waltham, MA, USA) using an EASY-nLC II liquid chromatography system. Tryptic peptides were subjected to liquid chromatography separation and electrospray ionization-based MS applying the same injected volumes in order to allow for label-free relative protein quantification between and within samples. Therefore, peptides were loaded on a self-packed analytical column (OD 360 μm, ID 100 μm, length 20 cm) filled with 3-μm diameter C18 particles (Maisch, Ammerbuch-Entringen, Germany) and eluted by a binary nonlinear gradient of 5 to 99% acetonitrile in 0.1% (vol/vol) acetic acid over 87 min with a flow rate of 300 nl/min. For MS analysis, a full scan in the Orbitrap with a resolution of 30,000 was followed by collision-induced dissociation (CID) of the 20 most abundant precursor ions. MS/MS experiments were acquired in the linear ion trap.

### MS data analysis.

Database searches against a database of PA PAO1 downloaded from the Pseudomonas Genome DB (48) on 6 October 2021 (5,587 entries) as well as Intensity-Based Absolute Quantification (iBAQ) were performed using MaxQuant (version 1.6.17.0) (106). MaxQuant enables high peptide identification rates, individualized p.p.b.-range mass accuracies, and proteome-wide protein quantification (107). Common laboratory contaminants and reversed sequences were included by MaxQuant. Search parameters were set as follows: trypsin/P-specific digestion with up to two missed cleavages, methionine oxidation and N-terminal acetylation as variable modification, match between runs with default parameters enabled. The false discovery rates (FDRs) of protein and peptide spectrum match (PSM) levels were set to 0.01. Two identified unique peptides were required for protein identification. Results were filtered for proteins quantified in at least two out of three biological replicates before statistical analysis. Here, protein from PAO1 grown in MM supplemented with 0.2 mM and 1 mM Pi were compared by a Student's *t* test applying a threshold *P* value of 0.01, which was based on all possible permutations. Proteins were considered differentially abundant if the log_2_ fold change was greater than 0.8. “ON/OFF proteins” were defined as being identified in all bioreplicates of one condition, whereas the protein was not identified in any replicate of the other condition.

### Bioinformatics.

Heatmap and hierarchical clustering analyses of the detected and differentially expressed proteins were made using the ComplexHeatmap version 2.9.3 package in R (108). Biological pathway enrichment analysis of differentially expressed proteins was performed using the BlastKOALA tool from the Kyoto Encyclopedia of Genes and Genomes (KEGG) (109). Fig. 4 to 10 were created using BioRender.com.

### Data availability.

The MS data reported in this publication have been deposited in the ProteomeXchange Consortium via the PRIDE partner repository (110) with data set identifier PXD033250.
